# Synthesis and physicochemical evaluation of fluorinated lipopeptide precursors of ligands for microbubble targeting

**DOI:** 10.3762/bjoc.17.45

**Published:** 2021-02-19

**Authors:** Masayori Hagimori, Estefanía E Mendoza-Ortega, Marie Pierre Krafft

**Affiliations:** 1 Institut Charles Sadron (CNRS), University of Strasbourg, 23 rue du Loess, 67034 Strasbourg CEDEX 2, France; 2 Faculty of Pharmaceutical Sciences, Mukogawa Women’s University, 11-68 Koshien Kyubancho, Nishinomiya 663-8179, Japan; 3 Graduate School of Biomedical Sciences, Nagasaki University, 1-7-1 Sakamoto, Nagasaki 852-8501, Japan

**Keywords:** adsorption at fluid interfaces, drug delivery, microbubble targeting, molecular imaging, monolayer, perfluoroalkylated lipopeptide, solid-phase peptide synthesis

## Abstract

Ligand-targeted microbubbles are focusing interest for molecular imaging and delivery of chemotherapeutics. Lipid–peptide conjugates (lipopeptides) that feature alternating serine–glycine (SG)*_n_* segments rather than classical poly(oxyethylene) linkers between the lipid polar head and a targeting ligand were proposed for the liposome-mediated, selective delivery of anticancer drugs. Here, we report the synthesis of perfluoroalkylated lipopeptides (*F*-lipopeptides) bearing two hydrophobic chains (C*_n_*F_2_*_n_*_+1_, *n* = 6, 7, 8, **1**–**3**) grafted through a lysine moiety on a hydrophilic chain composed of a lysine–serine–serine (KSS) sequence followed by 5 SG sequences. These *F*-lipopeptides are precursors of targeting lipopeptide conjugates. A hydrocarbon counterpart with a C_10_H_21_ chain (**4**) was synthesized for comparison. The capacity for the *F*-lipopeptides to spontaneously adsorb at the air/water interface and form monolayers when combined with dipalmitoylphosphatidylcholine (DPPC) was investigated. The *F*-lipopeptides **1**–**3** demonstrated a markedly enhanced tendency to form monolayers at the air/water interface, with equilibrium surface pressures reaching ≈7–10 mN m^−1^ versus less than 1 mN m^−1^ only for their hydrocarbon analog **4**. The *F*-lipopeptides penetrate in the DPPC monolayers in both liquid expanded (LE) and liquid condensed (LC) phases without interfacial film destabilization. By contrast, **4** provokes delipidation of the interfacial film. The incorporation of the *F*-lipopeptides **1**–**3** in microbubbles with a shell of DPPC and dipalmitoylphosphatidylethanolamine-PEG2000 decreased their mean diameter and increased their stability, the best results being obtained for the C_8_F_17_-bearing lipopeptide **3**. By contrast, the hydrocarbon lipopeptide led to microbubbles with a larger mean diameter and a significantly lower stability.

## Introduction

Various nano- and microsystems, including micelles, liposomes, and microbubbles, have been developed as imaging agents and to selectively deliver chemotherapeutics to tumor cells [1–6]. An increased specificity for tumor cells can be gained through ligand-mediated active targeting, which involves the use of targeting ligands, such as monoclonal antibodies, antibody fragments, proteins, peptides, and other small molecules, including vitamins and carbohydrates [7–8]. The targeting ligands are coupled to the surface of the carrier to selectively target tumor cells that overexpress a particular cell surface receptor [7,9–14]. To this aim, ligand–lipid conjugates have been developed in research and preclinical development for liposome targeting for decades. In particular, peptide ligands offer significant advantages, including efficient synthesis routes, versatility, and safety [15–17]. Various effective receptor-binding peptides have been identified by the phage display technology [18]. The peptides can be readily prepared through solid-phase peptide synthesis (SPPS), a highly reproducible method with minimal side reactions. Many peptide–lipid conjugates (lipopeptides) have been used as the amphiphilic components of drug delivery systems with anticancer properties, such as the tripeptide Arg–Gly–Asp (RGD) that binds to integrin α_v_β_3_, which is expressed on endothelial cells of various malignant tumors [15,19–22]. Other lipopeptides display cell penetrating properties, such as the transactivator of the transcription (TAT) peptide. Moreover, peptides being smaller than the antibodies generally induce a lower immunogenicity [15–17]. Micro- and nanocarriers are often covered by poly(ethylene glycol) (PEG) stealth coatings that significantly enhance blood circulation times by allowing them to evade immune detection. PEGs often play a key role in the design of the ligands as a spacer between the nanocarrier surface and the lipid. PEGs have, however, some shortcomings, such as a broad molecular weight distribution, large steric hindrance, and the occurrence of side reactions due to reactive groups introduced during PEG to lipids (or peptides) connecting reactions [23–25]. In particular, the PEG layer grafted on the surface of certain nanocarriers restricts the exposure of functional peptides [26–27].

Novel ligand-grafted lipids have been proposed for the preparation of functional drug carriers for clinical applications [25,28–29]. In order to alleviate the steric hindrance effect of PEG chains, a novel spacer consisting of alternating serine–glycine sequences (SG)*_n_* was introduced between the ligand and lipid within the molecular structure [30]. These lipopeptides have a discrete molecular weight and are produced by Fmoc (fluorenylmethoxycarbonyl protecting group) SPPS, a procedure in which the peptide chain is assembled stepwise while attached to an insoluble resin support, which allows the easy removal of the byproducts at each step by washing. The human epidermal growth factor receptor-2 (HER2)-targeting KCCYSL peptide–(SG)*_n_*–lipids in which the (SG)*_n_* (*n* = 3, 5, 7) sequence was used as a spacer allowed the reduction of the steric hindrance when compared to the conventional PEG2000 spacer [28]. Liposomes containing these peptide ligands dramatically increased cellular association in HER2-positive cells. Other lipids grafted to the RGD peptide and SG spacer were integrated in PEGylated liposomes and were efficiently associated with integrin α_v_β_3_-expressing Colon-26 cells [25].

**Scheme 1:**
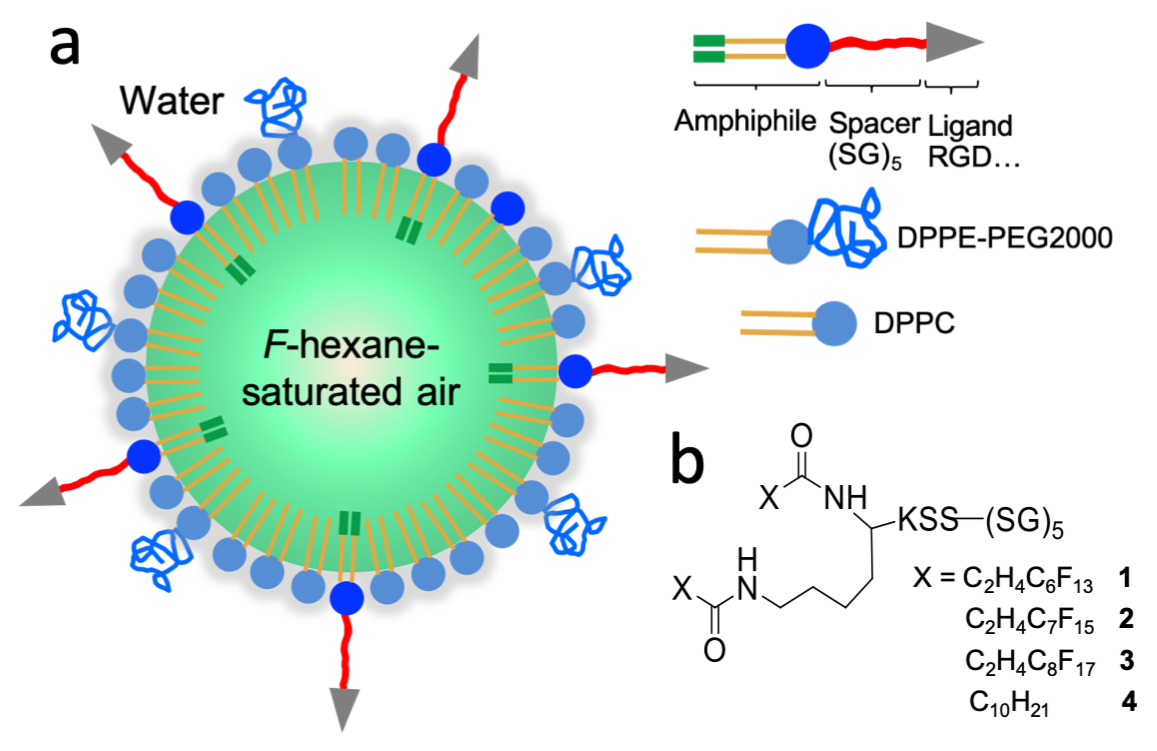
a) Schematic representation of a perfluorohexane-stabilized microbubble with a fluorinated lipopeptide anchored in its phospholipid shell and b) structures of the perfluoroalkylated lipopeptides **1**–**3** and of the hydrocarbon analog **4**.

One of our general objectives is to synthesize lipopeptides specifically designed for the incorporation in the phospholipid shell of medical microbubbles (MBs) (Scheme 1a). DPPC is widely used in the formulation of MBs, often in combination with a PEGylated dipalmitoylphosphatidylethanolamine (DPPE-PEG2000) that further enhances MB stability [31–33]. It is noteworthy that most of the phospholipid-stabilized MBs investigated in research and preclinical development are stabilized by a fluorocarbon (FC) gas [11,31,34]. FCs are known to contribute to MB stabilization through an osmotic effect [31]. In addition, FCs were also found to act as co-surfactants to the phospholipid molecules of the MB shell and strongly reduce its interfacial tension [35–36]. Recent studies have reported that the fluorine–fluorine interactions that develop between the FC and the MB shell component (e.g., fluorinated biomarkers [37] and fluorinated nanoparticles, including dendronized iron oxide nanoparticles [38] and nanodiamonds [39]) efficiently reinforce the interfacial film cohesion, thus enhancing the stability of the MBs. Various types of perfluoroalkylated amphiphiles have been reported that were designed for biomedical applications and display highly effective nanoemulsion and MB stabilizing characteristics [40–42].

In this work, we report the synthesis of a series of *F*-lipopeptides that are precursors of targeting lipopeptide conjugates and are specifically designed to be incorporated in the shell of phospholipid microbubbles. In a first step, the (SG)_5_KSS peptide chain is assembled stepwise using a Fmoc solid-phase peptide synthesis procedure. In a second step, the two perfluoroalkylated chains are grafted to the peptide chain through a lysine moiety. Next, the surface activity of the synthesized lipopeptides is investigated by assessing their ability to self-assemble into spontaneously adsorbed monolayers at the air/water interface and also to adsorb on a DPPC monolayer spread at the air/water interface. Finally, the size and stability characteristics of perfluorohexane (*F*-hexane)-stabilized microbubbles with DPPC/DPPE-PEG2000 shells and incorporating the new *F*-lipopeptides were determined and compared to those of reference MBs of similar phospholipid composition.

## Results and Discussion

### Synthesis and characterization of the lipid–peptide conjugates

Since the degree of fluorination of the hydrophobic chains of the lipid conditions the extent of fluorous interactions developed with the FC gas, we have selected various perfluoroalkyl chain lengths (C_6_F_13_, C_7_F_15_, and C_8_F_17_). The length of the (SG)*_n_* sequence was set to *n* = 5, which was found optimal in a previous report [28]. We synthetized three perfluoroalkylated double-chain peptide–lipid conjugates, (SG)_5_-KSS-K(C_2_H_4_-C*_n_*F_2_*_n_*_+1_)_2_ with *n* = 6 (**1)**, 7 (**2**) and 8 (**3**) (Scheme 1b). The hydrocarbon analog fitted with two C_10_H_21_ chains (**4**) was also prepared.

**Scheme 2:**
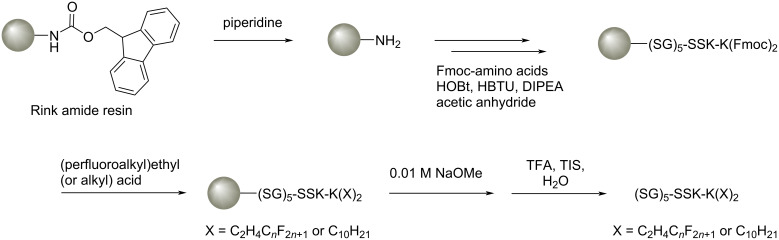
Solid-phase synthesis of *F*-lipopeptides **1**–**3** and hydrocarbon counterpart **4**.

The *F*-lipopeptide conjugates **1**–**3** and hydrocarbon analog **4** were obtained by a Fmoc solid-phase peptide synthesis method, in which the peptide sequence was stepwisely elongated, and eventually conjugated with the (perfluoroalkyl)ethyl acids (Scheme 2). After the cleavage from the resin, the Fmoc groups of the amino acids were removed, and the *F*-lipopeptides were purified using a dialysis membrane. According to mass spectrometry, FTIR, and HPLC analysis, the products **1**–**4** had high purity (>99%) (Supporting Information File 1, Figures S1–S12).

### Behavior of lipid-peptide conjugates at the air/water interface

**Figure 1:**
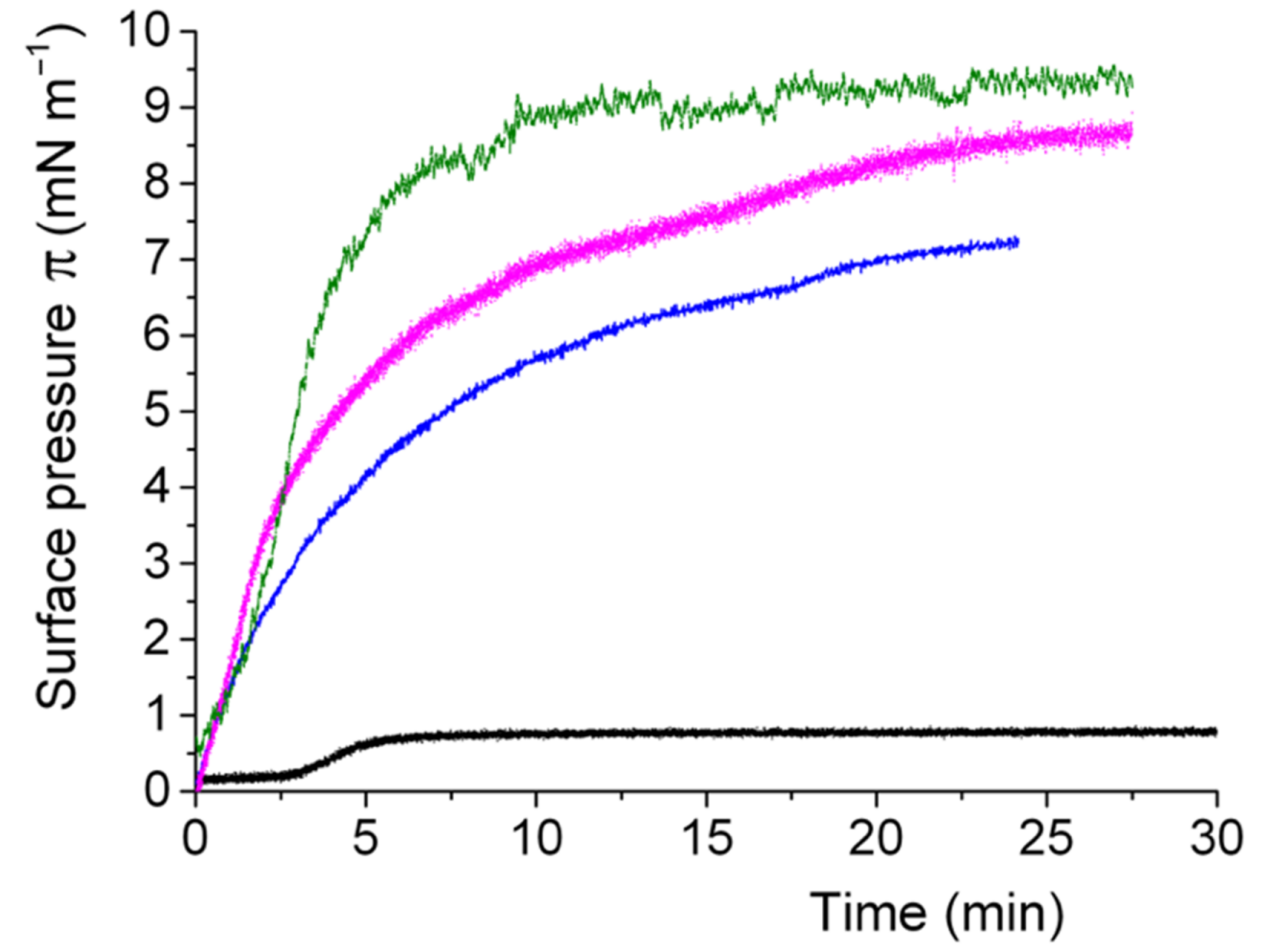
Adsorption kinetics of perfluoroalkylated lipopeptides **1**–**3** and the hydrocarbon analog **4** at the air/water interface (25 °C). Variation of surface pressure π as a function of time for **1** (blue), **2** (magenta), **3** (green), and **4** (black).

**Spontaneous adsorption of lipid–peptide conjugates at the air/water interface.** In order to investigate the capacity for *F*-lipopeptides **1**–**3** to spontaneously self-assemble into ordered monolayers at the air/water interface, we injected a solution of each peptide–lipid conjugate in DMSO into the aqueous sub-phase of an adsorption trough. The variation of the surface pressure π was measured over time at 25 °C (Figure 1). In all cases, π increased, reflecting a progressive adsorption at the interface, then reached a plateau, and stabilized at the equilibrium surface pressure (π_eq_). The adsorption kinetics demonstrate that the *F*-lipopeptides formed stable monolayers at the interface. The π_eq_ values increased with the degree of fluorination of the *F*-lipopeptides (≈7.2 mN m^−1^ for **1**, 8.6 mN m^−1^ for **2**, and 9.4 mN m^−1^ for **3**; ±0.5 mN m^−1^), reflecting their increasingly hydrophobic character. By contrast, the hydrocarbon analog **4** adsorbed considerably less, reaching a π_eq_ value of only 0.7 mN m^−1^. The adsorption of the *F*-lipopeptides is also much faster than that of the hydrocarbon compound (characteristic time of adsorption τ ≈ 0.5 min for **1**–**3** versus ≈2.5 min for **4**).

**Figure 2:**
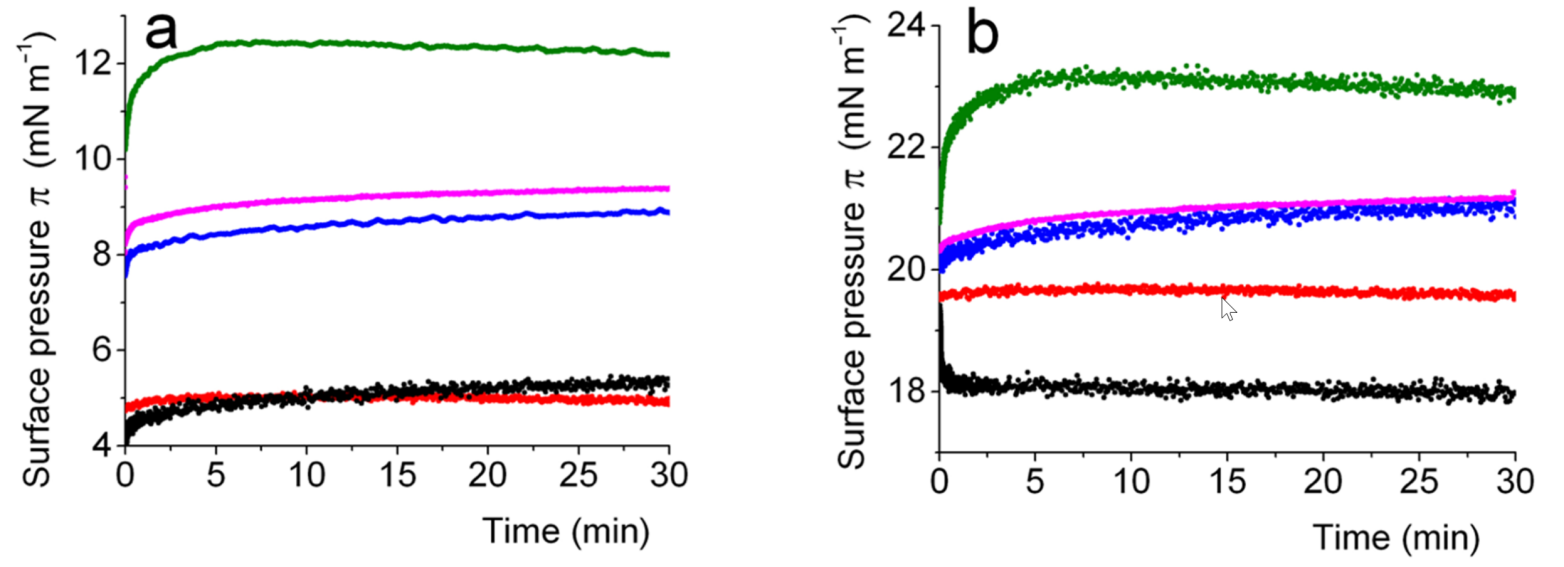
Adsorption of perfluoroalkylated lipopeptides **1**–**3** and hydrocarbon analog **4** on DPPC monolayers spread at the air/water interface a) in the liquid expanded (LE) and b) in the liquid condensed (LC) phases (25 °C). Variation of the surface pressure π as a function of time for a DPPC monolayer (red), and after injection of the lipopeptides in the aqueous sub-phase of a DPPC monolayer for *F*-lipopeptides: **1** (blue), **2** (magenta), **3** (green), and hydrocarbon analog **4** (black).

**Adsorption of lipid–peptide conjugates on a phospholipid monolayer spread at the air/water interface.** DPPC is widely used in the formulation of liposomes and microbubbles [31,33]. In order to investigate the ability of the *F*-lipopeptides to form mixed monolayers with DPPC at the air/water interface, the *F*-lipopeptides were injected in the aqueous sub-phase of a Langmuir monolayer of DPPC. Depending on the volume of the DPPC solution deposited, the monolayer is either in the liquid expanded (LE, 5 mN m^−1^) or in the liquid condensed (LC, 19 mN m^−1^) phase (Figure 2a,b). In the LE phase, the π values of the *F*-lipopeptides were significantly higher than that of the DPPC monolayer (Figure 2a) and remained stable over time, which means that the lipopeptides are inserted in the DPPC monolayer. On the other hand, the injection of the hydrocarbon analog **4** was not followed by an increase of π, which suggests that **4** is not adsorbed in the DPPC monolayer. In the LC phase, π_eq_ is ≈19 mN m^−1^ for DPPC alone. We observed that π_eq_ increased significantly after the injection of the *F*-lipopeptides, reflecting the insertion in the DPPC monolayer. The higher the degree of fluorination, the higher the amount inserted, with a maximal efficiency observed for lipopeptide **3** (C_8_F_17_). However, the behavior of the *F*-lipopeptide **2** with an odd number of carbon atoms (C_7_F_15_) is closer to that of **1** (C_6_F_13_) than to that of **3**, and not intermediate, as it could have been expected. It may possibly be that this comportment can be ascribed to odd/even effects, which are known to impact on the adsorption process of surfactants [43]. For example, surfactants with an odd number of carbon atoms in their alkyl chain were less effective for emulsifying liquid crystals than those with an even number of carbons [44]. The behavior of the hydrocarbon lipopeptide **4** was markedly different, with a decrease of the surface pressure over time, and a much lower π_eq_. This not only means that the hydrocarbon analog is not recruited at the interface, but also that there is a significant loss of molecules, and that contact of the hydrocarbon lipid with the DPPC monolayer causes a delipidation of the interface.

### Generation of microbubbles from combinations of DPPC and lipid–peptide conjugates

**Figure 3:**
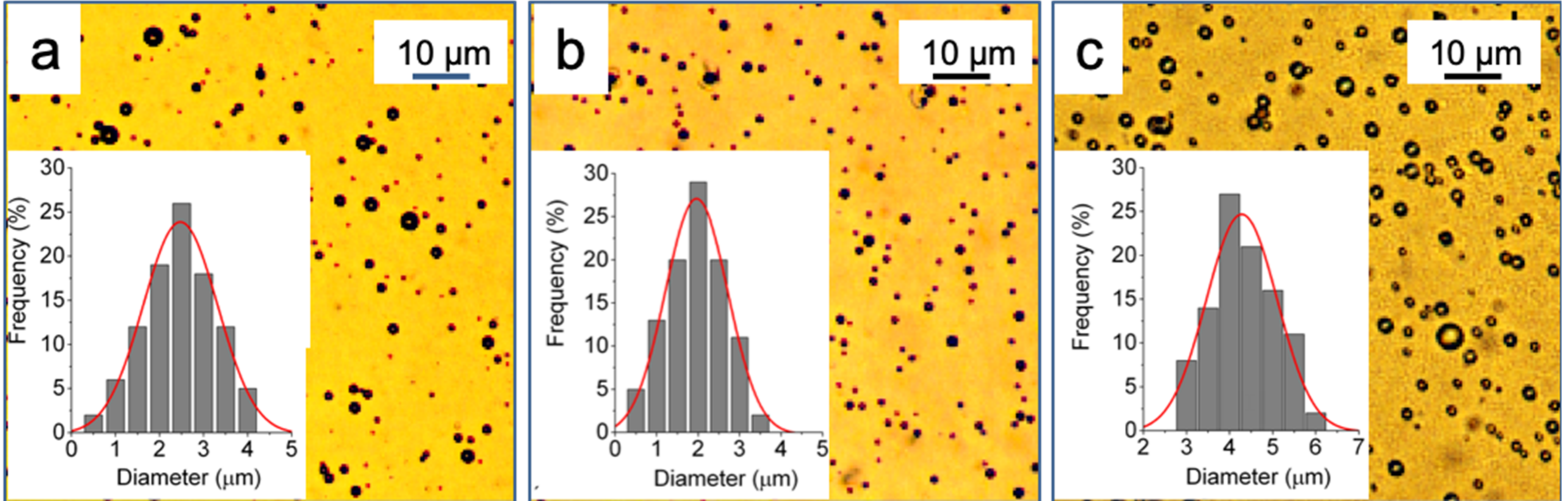
Optical micrographs and corresponding size distribution of the perfluorohexane-stabilized microbubbles with a shell of a) DPPC, b) DPPC/*F*-lipopeptide **3**, and c) DPPC/hydrocarbon analog **4**.

Next, we have investigated whether microbubbles incorporating the lipopeptides in the shell could be produced and what their effect on the size characteristics and stability of the resulting MBs would be. We therefore selected DPPC and DPPE-PEG2000 as the main MB shell components. The PEGylated phospholipid is often used in MB formulations to increase MB half-lives. The microbubbles were prepared by mechanical agitation using a Vialmix shaker and were characterized by optical microscopy immediately after the preparation and over time. The results show that the incorporation of *F*-lipopeptides **1**–**3** led to MBs that are somewhat smaller than those made from DPPC alone (e.g., 1.9 ± 0.6 μm with *F*-lipopeptide **3** versus 2.5 ± 0.8 μm without, Figure 3a,b). Microbubbles with similar mean diameters were obtained with the two other *F*-lipopeptides. By contrast, the incorporation of the hydrocarbon analog **4** led to a marked increase in the mean MB diameter (4.3 ± 0.9 μm, Figure 3c).

**Figure 4:**
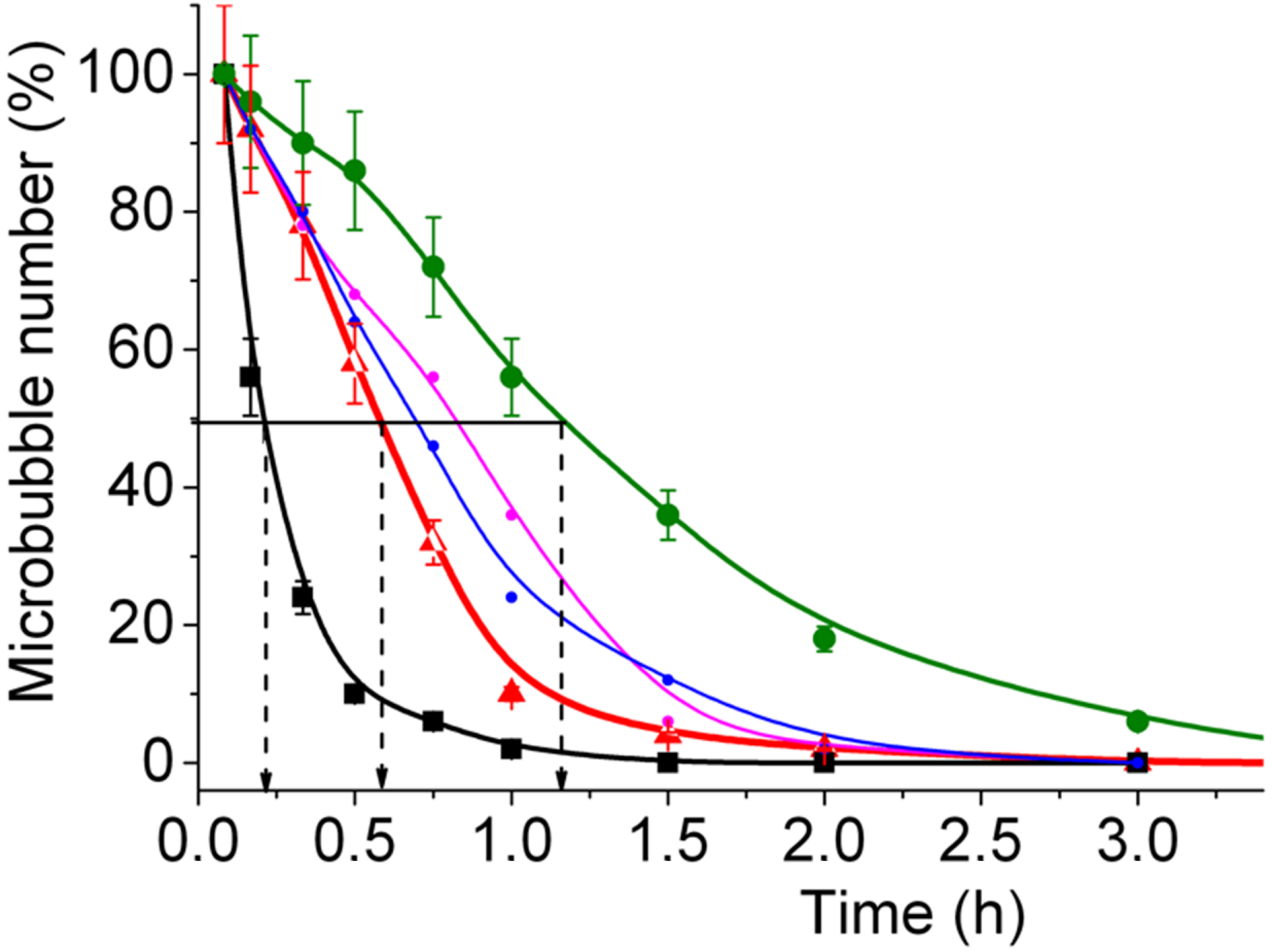
Half-lives of microbubbles (25 °C) containing *F*-lipopeptides **1**–**3** and hydrocarbon analog **4**.

Finally, we have investigated the stability of the microbubbles over time at room temperature. The MBs containing the *F*-lipopeptides were found to be more stable than the reference DPPC/DPPE-PEG2000 MBs (Figure 4). The most stable MBs were those containing the *F*-lipopeptide **3** with a half-life of 1.1 ± 0.2 h, as compared to 0.6 ± 0.2 for DPPC MBs. MBs incorporating the *F*-lipopeptides **1** and **2** displayed intermediate half-lives.

The reduction in size of the MBs incorporating the *F*-lipopeptides compared to those incorporating the hydrocarbon compound **4** can be explained by the faster diffusion of the former lipopeptides to the interface and by their larger value of π_eq_, hence a lower surface tension at the bubble surface. The enhanced MB stability over time and the fact that the half-life increases with fluorocarbon chain length support the view that stabilizing interactions develop between the *F*-lipopeptide and perfluorohexane in the bubble’s interfacial film.

## Conclusion

A series of lipopeptides carrying C*_n_*F_2_*_n_*_+1_ chains (*n* = 6, 7, 8, **1**–**3**) or C_10_H_21_ chains (**4**) grafted through a lysine moiety on a peptide chain composed of a KSS sequence followed by 5 SG sequences were synthesized by Fmoc solid-phase peptide synthesis. The investigation of the physicochemical properties of these lipopeptides at the air/water interface demonstrates that the fluorination substantially improves the surface-active properties. In our experimental conditions, the fluorination enables a significantly larger and faster adsorption, both at the surface of water and on the DPPC monolayers in both the LE and LC states. By contrast, the adsorption of the hydrocarbon analog is only possible when the phospholipid monolayer is in the LE state, whilst its adsorption in the LC state is not only prohibited, but even provokes a delipidation of the interface. The incorporation of the perfluoroalkylated lipopeptides in the phospholipid shells of perfluorohexane-stabilized microbubbles significantly reduces their mean size and increases their stability. By contrast, larger bubbles with shorter half-lives are obtained with the hydrocarbon analog. Our results establish that a fluorination of the precursors of targeting ligand–peptide conjugates can considerably facilitate microbubble generation due to faster diffusion to the air/water interface, and augment their stability through interfacial fluorine–fluorine interactions.

## Experimental

**Materials**. We purchased Fmoc-protected amino acids, *N*,*N*-dimethylformamide (DMF), dichloromethane (DCM), methanol, 1-hydroxybenzotriazole (HOBT), 2-(1*H*-benzotriazole-1-yl)-1,1,3,3-tetramethyluronium hexafluorophosphate (HBTU), *N*,*N*-diisopropylethylamine (DIPEA), piperidine, acetic anhydride, trifluoroacetic acid (TFA), triisopropylsilane (TIS), Rink Amide AM resin (4-(2’,4’-dimethoxyphenyl-Fmoc-aminomethyl)phenoxyacetamido-aminomethyl resin, 100–200 mesh), and Tube-O-DIALYZER™ mini dialysis system (MWCO 1K) from Merck (Darmstadt, Germany). 1,2-Dipalmitoylphosphatidylcholine (DPPC, >99%) and 1,2-dipalmitoyl-*sn*-glycero-3-phosphoethanolamine-*N*-[methoxy(polyethylene glycol)-2000] (DPPE-PEG2000, >99%) were purchased from Avanti Polar Lipids (Alabaster, AL, USA) and used without further purification. Perfluorohexane came from Fluorochem (>98%). A HEPES (*N*-2-(hydroxyethyl)piperazine-*N*′-(2-ethanesulfonic acid), powder, 99.5%, Corning, NY) buffer solution (20 mM) in 150 mM NaCl was prepared and adjusted to pH 7.4 using 0.1 N NaOH. Chloroform (99.4%) was purchased from VWR (Avantor, Fontenay-sous-Bois). Ultrapure water was obtained from a Milli-Q (Millipore Corp.) system (surface tension: 72.1 mN m^−1^ at 20 °C, resistivity: 18.2 MΩ cm). Mass spectra (MS) and HRMS were performed using a JMS-700 spectrometer (JEOL, Japan). RP-HPLC chromatograms were recorded on a Prominence system (Shimadzu, Japan). FTIR spectra were recorded on IRAffinity-1 (Shimadzu, Japan) spectrometer.

**General procedure for the synthesis of perfluoroalkylated lipopeptides**. All *F*-lipopeptides (SG)_5_-KSS-K(C_2_H_4_-C*_n_*F_2_*_n_*_+1_)_2_ with *n* = 6 (**1**), 7 (**2**), 8 (**3**) and hydrocarbon analog (SG)_5_-KSS-K(C_10_H_21_)_2_ were synthesized using an Fmoc solid-phase peptide synthesis (SPPS) method. Rink Amide AM resin (0.1 mmol) in a 10 mL column was suspended in 5 mL of DMF and swollen overnight. After washing with DMF (3 × 2 mL), the Fmoc groups of the Rink amide AM resin were activated with 20% of piperidine in DMF (2 mL) for 20 min. After washing with DMF (3 × 2 mL), Fmoc-Ser(*t*-Bu)-OH (3 equiv) as the first Fmoc-amino acid and the mixture of HBTU, HOBT, and DIPEA (3 equiv/3 equiv/6 equiv) in DMF were added to the resin and shaken for 30 min. The reaction was monitored using a Kaiser test based on the reaction of ninhydrin. After washing with DMF (3 × 2 mL) and DCM (3 × 2 mL), 25% of acetic anhydride in DCM (2 mL) was added for capping the unreacted amino acids and the mixture was shaken for 5 min. In a similar manner, each of the peptide chains was elongated by coupling Fmoc-Ser(*t*-Bu)-OH (3 equiv), Fmoc-Gly-OH (3 equiv) and Fmoc-Lys(Boc)-OH (3 equiv) to the Rink Amide AM resin. After introducing Fmoc-Lys(Fmoc)-OH as the terminal amino acid, the Fmoc groups of Fmoc-Lys(Fmoc)-OH were activated with 2 × 20% of piperidine in DMF (2 mL) for 20 min, and the coupling reaction with perfluoroalkylated acids (3 equiv) or alkyl acid (3 equiv) was performed 3 times with HBTU/HOBT/DIPEA (6 equiv/6 equiv/12 equiv) for 3 h. After capping the reaction with 25% of acetic anhydride in DCM (2 mL), the column was washed with DCM (3 × 2 mL), DMF (3 × 2 mL), and methanol (3 × 2 mL), and dried overnight. A solution of 2.5 mL of TFA/TIS/H_2_O 94:2.5:2.5 (v/v/v) was added to the column for cleaving the compound from the resin, and the reaction was performed for 3 h. The TFA/TIS/H_2_O solution including the crude product was collected in a 50 mL Erlenmeyer flask. The column was washed 3 times with TFA (1.5 mL), and the washing solutions were combined. After drying the solution with argon gas, the residue was washed with 20 mL of diethyl ether. The product was collected by filtration and purified by dialysis using a Tube-O-DIALYZER™ mini dialysis system. The purity of the final products was analyzed by a high-performance liquid chromatography (HPLC) system using a reversed-phase column (COSMOSIL 5C18-AR-II 4.6 × 250 mm) with water and acetonitrile 20:80 (v/v) at a flow rate of 0.5 mL/min.

(SG)_5_-KSS-K(C_2_H_4_-C_6_F_13_)_2_ (**1**) was synthesized according to the general procedure using 4,4,5,5,6,6,7,7,8,8,9,9,9-tridecafluorononanoic acid. Yield: 121 mg (6.3%); FABMS (*m*/*z*): 1916 [M + H]^+^; HRMS (*m*/*z*): [M + H]^+^ calcd for C_61_H_84_F_26_N_17_O_23_, 1916.5511; found: 1916.5509; purity (retention time): >99% (13.8 min).

(SG)_5_-KSS-K(C_2_H_4_-C_7_F_15_)_2_ (**2**) was synthesized according to the general procedure using 4,4,5,5,6,6,7,7,8,8,9,9,10,10,10-pentadecafluorodecanoic acid. Yield: 95 mg (4.7%); FABMS (*m*/*z*): 2016 [M + H]^+^; HRMS (*m*/*z*): [M + H]^+^ calcd for C_63_H_84_F_30_N_17_O_23_, 2016.5447; found, 2016.5448; purity (retention time): >99% (14.8 min).

(SG)_5_-KSS-K(C_2_H_4_-C_8_F_17_)_2_ (**3**) was synthesized according to the general procedure using 4,4,5,5,6,6,7,7,8,8,9,9,10,10,11,11,11-heptadecafluoroundecanoic acid. Yield: 111 mg (5.2%); FABMS (*m*/*z*): 2116 [M + H]^+^; HRMS (*m*/*z*): [M + H]^+^ calcd for C_65_H_84_F_34_N_17_O_23_, 2116.5383; found, 2116.5381; purity (retention time): >99% (13.8 min).

(SG)_5_-KSS-K(C_10_H_21_)_2_ (**4**) was synthesized according to the general procedure using undecanoic acid. Yield: 70 mg (4.7%); FABMS (*m*/*z*): 1504 [M + H]^+^; HRMS (*m*/*z*): [M + H]^+^ calcd for C_65_H_118_N_17_O_23_,1504.8586; found, 1504.8585; purity (retention time): >99% (13.9 min).

**Adsorption kinetics of lipopeptides at the air/water interface**. The experiments were conducted in a home-made Teflon adsorption trough (11.9 × 5.0 × 0.3 cm^3^) filled with HEPES buffer (pH 7.4). The surface pressure π was measured using the Wilhelmy plate method. The temperature was maintained at 25 ± 0.5 °C. For the spontaneous formation of monolayers (Gibbs films) at the air/water interface, 50 μL of the solutions of the lipopeptides **1**–**4** in DMSO (1 mmol L^−1^) were injected into the aqueous phase. For the experiment concerning the adsorption of lipopeptides on a DPPC Langmuir monolayer, a solution of DPPC in chloroform (1 mmol L^−1^) was deposited on the surface of the aqueous phase. Depending on the volume deposited (9 μL or 18 μL), DPPC monolayers were obtained in the liquid expended or in the liquid condensed phase. Ten minutes were allowed to evaporate chloroform. Then, 50 μL of the solutions (1 mmol L^−1^) of the lipopeptides in DMSO were injected in the aqueous sub-phase and the surface pressure was monitored over time. Three separate experiments were conducted for each lipopeptide. The error in the surface pressure measurements was ±0.5 mN m^−1^.

**Preparation and characterization of lipopeptide-containing microbubbles.** DPPC (50 mmol L^−1^) and DPPE-PEG2000 (DPPC/DPPE-PEG2000 molar ratio 9:1) were dispersed in a HEPES buffer solution (0.9 mL) by magnetic stirring for 3–6 h at 50 °C. Fifty µL of the lipopeptide solution in DMSO were injected into the phospholipid dispersion and subjected to agitation/amalgamation using a Vialmix^®^ device (2 cycles of 45 s, Lantheus Medical Imaging N. Billerica, MA) at room temperature and under *F*-hexane-saturated N_2_ at room temperature (for details, see [39]). The resulting foam was immediately diluted with 5 mL of HEPES buffer. Size fractionation of the microbubbles was achieved by flotation for 60 min. Reference microbubbles shelled with DPPC/DPPE-PEG2000 were prepared using the same protocol. Two to three droplets of the bubble dispersion were placed into a concave glass slide, covered with a glass slide and observed with a Nikon Eclipse 90i microscope (transmission mode, Nikon Instruments Europe, Amsterdam, The Netherlands). A rapid image acquisition was achieved using a Lumenera Infinity 2 charge-coupled device (CCD) camera (Lumenera, Ottawa, Canada). The bubble mean diameter and distribution width after preparation and upon time were determined on 5–10 slides using Fiji (an open-source image processing package [45]) and the standard deviations were calculated using Origin9 (OriginLab Corp. Northampton, MA, USA).

## Supporting Information

File 1 Mass spectrometry and FTIR data as well as RP-HPLC chromatograms of lipopeptides **1**–**4**.
