# Supplementary material for: Synthesis and physicochemical evaluation of fluorinated lipopeptide precursors of ligands for microbubble targeting
Source: Beilstein J Org Chem. 2021 Feb 19;17:511–8. doi: 10.3762/bjoc.17.45 (PMC7934786; doi:10.3762/bjoc.17.45)
Supplement: File 1 — Mass spectrometry and FTIR data as well as RP-HPLC chromatograms of lipopeptides 1–4. [file Beilstein_J_Org_Chem-17-511-s001.pdf]

BEILSTEIN JOURNAL OF ORGANIC CHEMISTRY

# Supporting Information

for

# Synthesis and physicochemical evaluation of fluorinated lipopeptide precursors of ligands for microbubble targeting

Masayori Hagimori, Estefanía E. Mendoza-Ortega and Marie Pierre Krafft

*Beilstein J. Org. Chem.* **2021,** *17,* 511–518. doi:10.3762/bjoc.17.45

## Mass spectrometry and FTIR data as well as RP-HPLC chromatograms of lipopeptides 1–4

License and Terms: This is a supporting information file under the terms of the Creative Commons Attribution License (https://creativecommons.org/licenses/by/4.0). Please note that the reuse, redistribution and reproduction in particular requires that the author(s) and source are credited and that individual graphics may be subject to special legal provisions.
The license is subject to the *Beilstein Journal of Organic Chemistry* terms and conditions: (https://www.beilstein-journals.org/bjoc/terms)

# Table of contents


1. Instrumentation S2

2. Figure S1: Mass spectrometry data of $(SG)_5$-KSS-K$(C_2H_4$-$C_6F_{13})_2$ **1** S2

3. Figure S2: RP-HPLC chromatogram of $(SG)_5$-KSS-K$(C_2H_4$-$C_6F_{13})_2$ **1** S3

4. Figure S3: FTIR spectrum of $(SG)_5$-KSS-K$(C_2H_4$-$C_6F_{13})_2$ **1** S3

5. Figure S4: Mass spectrometry data of $(SG)_5$-KSS-K$(C_2H_4$-$C_7F_{15})_2$ **2** S4

6. Figure S5: RP-HPLC chromatogram of $(SG)_5$-KSS-K$(C_2H_4$-$C_7F_{15})_2$ **2** S4

7. Figure S6: FTIR spectrum of $(SG)_5$-KSS-K$(C_2H_4$-$C_7F_{15})_2$ **2** S5

8. Figure S7: Mass spectrometry data of $(SG)_5$-KSS-K$(C_2H_4$-$C_8F_{17})_2$ **3** S5

9. Figure S8: RP-HPLC chromatogram of $(SG)_5$-KSS-K$(C_2H_4$-$C_8F_{17})_2$ **3** S6

10. Figure S9: FTIR spectrum of $(SG)_5$-KSS-K$(C_2H_4$-$C_8F_{17})_2$ **3** S6

11. Figure S10: Mass spectrometry data of $(SG)_5$-KSS-K$(C_{10}H_{21})_2$ **4** S7

12. Figure S11: RP-HPLC chromatogram of $(SG)_5$-KSS-K$(C_{10}H_{21})_2$ **4** S7

13. Figure S12: FTIR spectrum of $(SG)_5$-KSS-K$(C_{10}H_{21})_2$ **4** S8

**Instrumentation:**

Mass spectra (MS) and HRMS were performed using a JMS-700 spectrometer (JEOL, Japan). RP-HPLC chromatograms were recorded on a Prominence system (Shimadzu, Japan). FTIR spectra were recorded on IRAffinity-1 (Shimadzu, Japan).

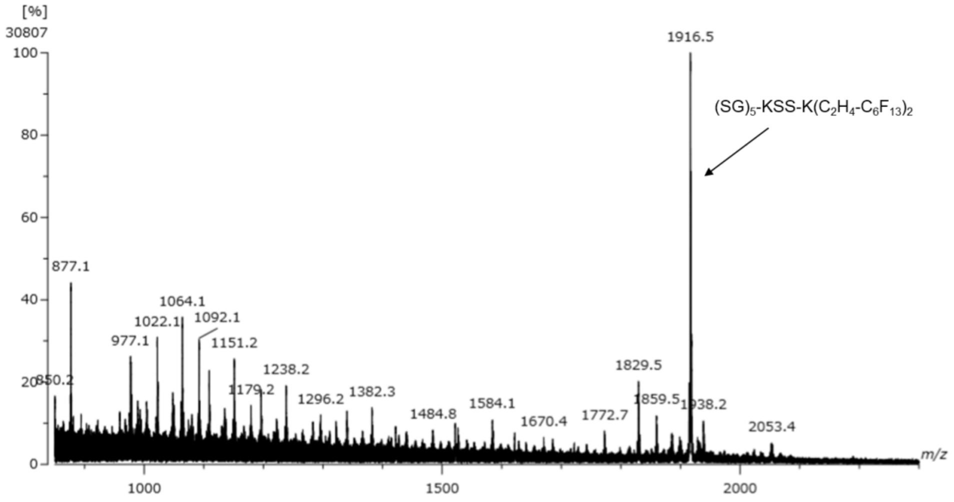


**Figure S1:** Mass spectrometry data of $(SG)_5$-KSS-K$(C_2H_4$-$C_6F_{13})_2$ **1**. Exact mass of the $(SG)_5$-KSS-K$(C_2H_4$-$C_6F_{13})_2$ $[M + H]^+$ was calculated 1916.5511 and found 1916.5509.

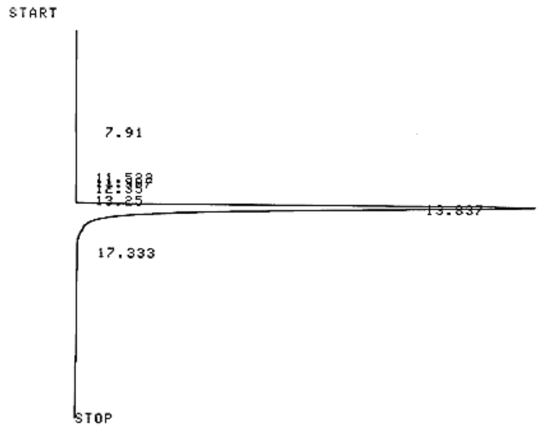


CHROMATOGRAM 1 MEMORIZED

CHROMATOPAC C-R6A FILE 0
SAMPLE NO 0 METHOD 41
REPORT NO 9452

| PKNO | TIME | AREA | MK | IDNO | CONC | NAME |
|---|---|---|---|---|---|---|
| 1 | 7.91 | 10166 | | | 0.0381 | |
| 2 | 11.522 | 11036 | | | 0.0413 | |
| 3 | 11.987 | 5531 | V | | 0.0207 | |
| 4 | 13.25 | 3779 | | | 0.0141 | |
| 5 | 13.837 | 26645216 | S | | 99.7371 | |
| 6 | 17.333 | 39727 | T | | 0.1487 | |
| | TOTAL | 26715456 | | | 100 | |

**Figure S2:** RP-HPLC chromatogram of (SG)$_5$-KSS-K($C_2H_4$-$C_6F_{13}$)$_2$ **1**. HPLC conditions: the column was a COSMOSIL 5C18-AR-II 4.6 mm × 250 mm, flow rate was 0.5 mL/min, UV excitation at 220 nm, mobile phase systems were $CH_3CN/H_2O$ 80:20.

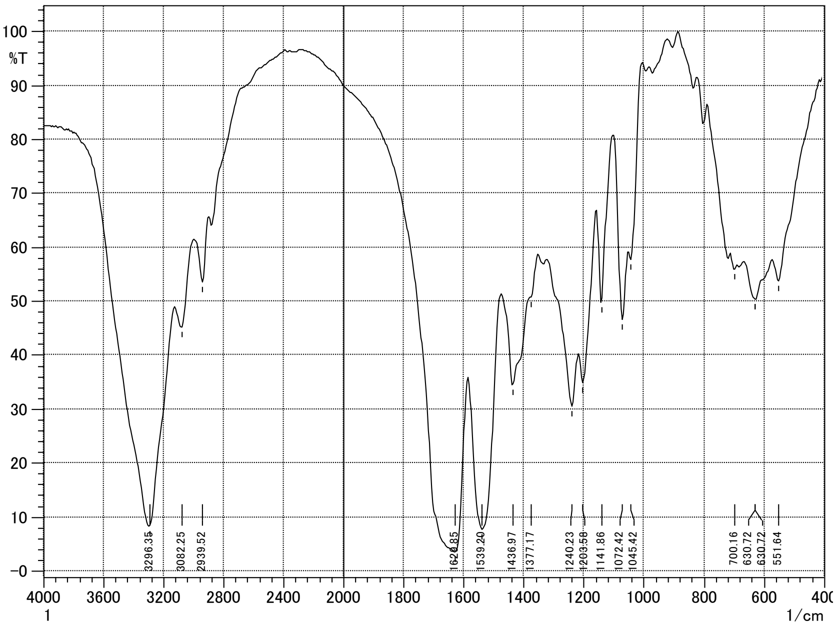


**Figure S3:** FTIR spectrum of (SG)$_5$-KSS-K($C_2H_4$-$C_6F_{13}$)$_2$ **1**.

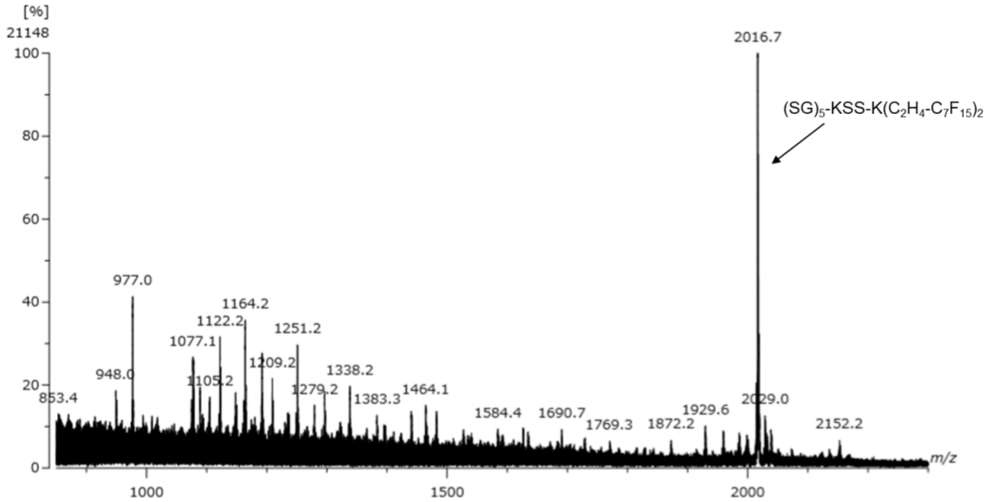


**Figure S4:** Mass spectrometry data of (SG)$_5$-KSS-K($C_2H_4$-$C_7F_{15}$)$_2$ **2**. Exact mass of the (SG)$_5$-KSS-K($C_2H_4$-$C_7F_{15}$)$_2$ [M + H]$^+$ was calculated 2016.5447 and found 2016.5448.

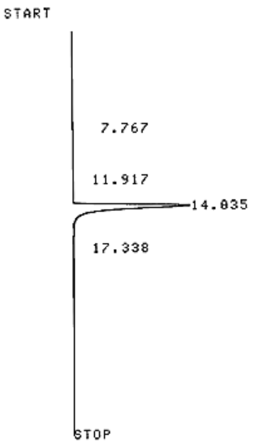


CHROMATOGRAM 6 MEMORIZED

CHROMATOPAC C-R6A FILE 0
SAMPLE NO 0 METHOD 41
REPORT NO 9457

| PKNO | TIME | AREA | MK | IDNO | CONC | NAME |
|---|---|---|---|---|---|---|
| 1 | 7.767 | 2274 | | | 0.0372 | |
| 2 | 11.917 | 1079 | | | 0.0177 | |
| 3 | 14.035 | 6079956 | | | 99.4435 | |
| 4 | 17.338 | 30672 | | | 0.5017 | |
| | TOTAL | 6113980 | | | 100 | |

ATTEN(0)=9
ANAL 0
*ERROR* 2:ILLEGAL QUANTITY

**Figure S5:** RP-HPLC chromatogram of (SG)$_5$-KSS-K($C_2H_4$-$C_7F_{15}$)$_2$ **2**. HPLC conditions: the column was a COSMOSIL 5C18-AR-II 4.6 mm × 250 mm, flow rate was 0.5 mL/min, UV excitation at 220 nm, mobile phase systems were $CH_3CN/H_2O$ 80:20.

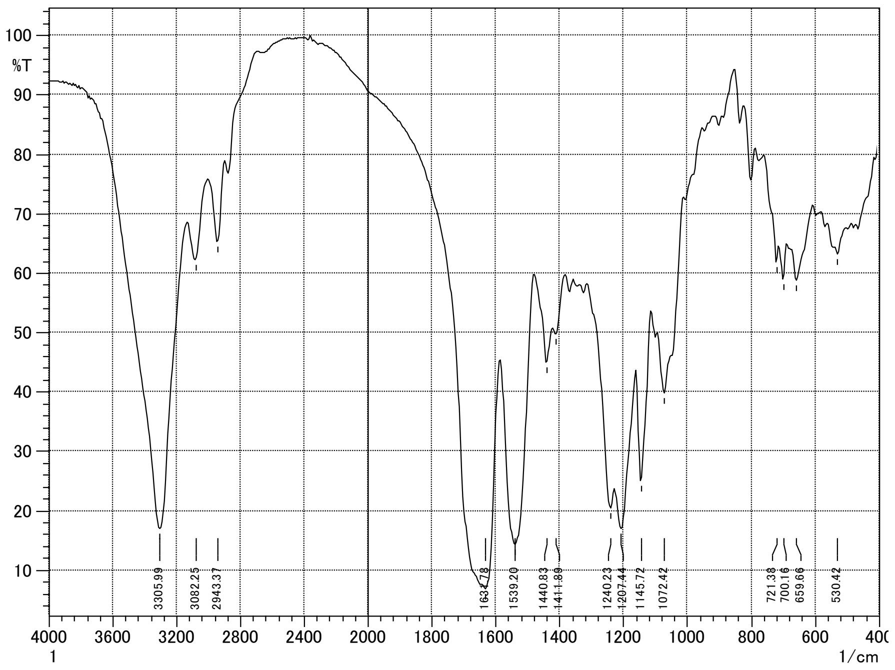


**Figure S6:** FTIR spectrum of $(SG)_5$-KSS-K$(C_2H_4$-$C_7F_{15})_2$ **2**.

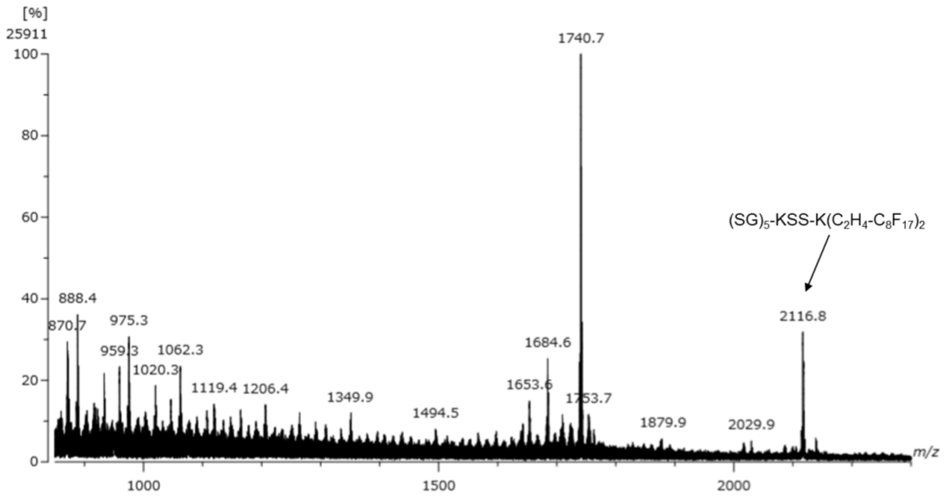


**Figure S7:** Mass spectrometry data of $(SG)_5$-KSS-K$(C_2H_4$-$C_8F_{17})_2$ **3**. Exact mass of the $(SG)_5$-KSS-K$(C_2H_4$-$C_8F_{17})_2$ $[M + H]^+$ was calculated 2116.5383 and found 2116.5381.

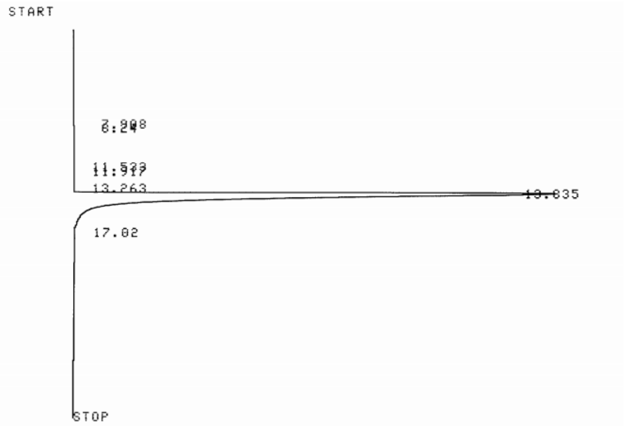


CHROMATOGRAM 3 MEMORIZED

CHROMATOPAC C-R6A FILE 0
SAMPLE NO 0 METHOD 41
REPORT NO 9454

| PKNO | TIME | AREA | MK | IDNO | CONC | NAME |
|---|---|---|---|---|---|---|
| 1 | 7.908 | 7909 | | | 0.0261 | |
| 2 | 8.24 | 3933 | V | | 0.013 | |
| 3 | 11.533 | 2571 | | | 0.0085 | |
| 4 | 11.917 | 3333 | V | | 0.011 | |
| 5 | 13.835 | 30275266 | S | | 99.8899 | |
| 6 | 17.02 | 15622 | T | | 0.0515 | |
| | TOTAL | 30308630 | | | 100 | |

**Figure S8:** RP-HPLC chromatogram of $(SG)_5$-KSS-K$(C_2H_4$-$C_8F_{17})_2$ **3**. HPLC conditions: the column was a COSMOSIL 5C18-AR-II 4.6 mm × 250 mm, flow rate was 0.5 mL/min, UV excitation at 220 nm, mobile phase systems were $CH_3CN/H_2O$ 80:20.

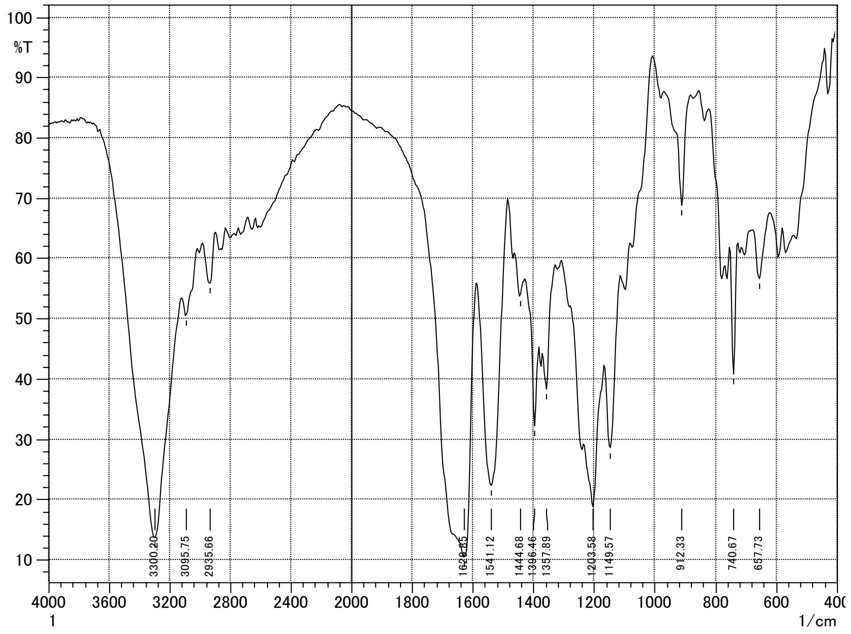


**Figure S9:** FTIR spectrum of $(SG)_5$-KSS-K$(C_2H_4$-$C_8F_{17})_2$ **3**.

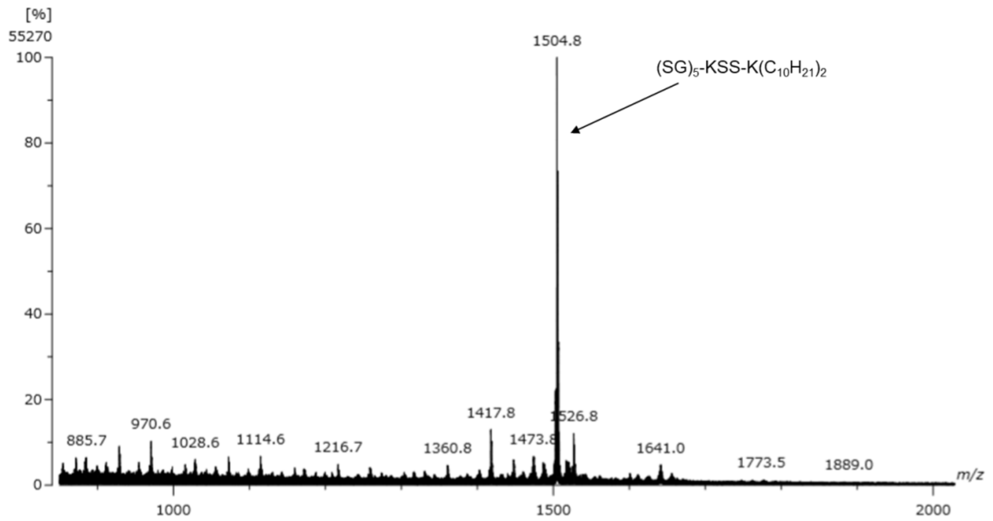


**Figure S10:** Mass spectrometry data of $(SG)_5$-KSS-K$(C_{10}H_{21})_2$ **4**. Exact mass of the $(SG)_5$-KSS-K$(C_{10}H_{21})_2$ $[M + H]^+$ was calculated 1504.8586 and found 1504.8585.

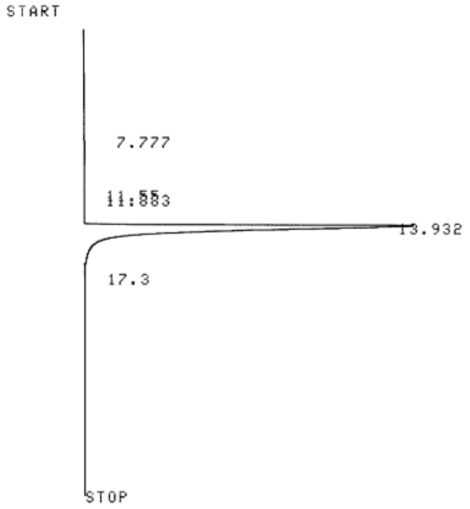


CHROMATOGRAM 2 MEMORIZED

CHROMATOPAC C-R6A FILE 0
SAMPLE NO 0 METHOD 41
REPORT NO 9459

| PKNO | TIME | AREA | MK | IDNO | CONC | NAME |
|---|---|---|---|---|---|---|
| 1 | 7.777 | 3043 | | | 0.0194 | |
| 2 | 11.55 | 3839 | | | 0.0245 | |
| 3 | 11.883 | 2677 | V | | 0.0171 | |
| 4 | 13.932 | 15635736 | S | | 99.8026 | |
| 5 | 17.3 | 21370 | T | | 0.1364 | |
| | TOTAL | 15666662 | | | 100 | |

**Figure S11:** RP-HPLC chromatogram of $(SG)_5$-KSS-K$(C_{10}H_{21})_2$ **4**. HPLC conditions: the column was a COSMOSIL 5C18-AR-II 4.6 mm × 250 mm, flow rate was 0.5 mL/min, UV excitation at 220 nm, mobile phase systems were $CH_3CN/H_2O$ 80:20.

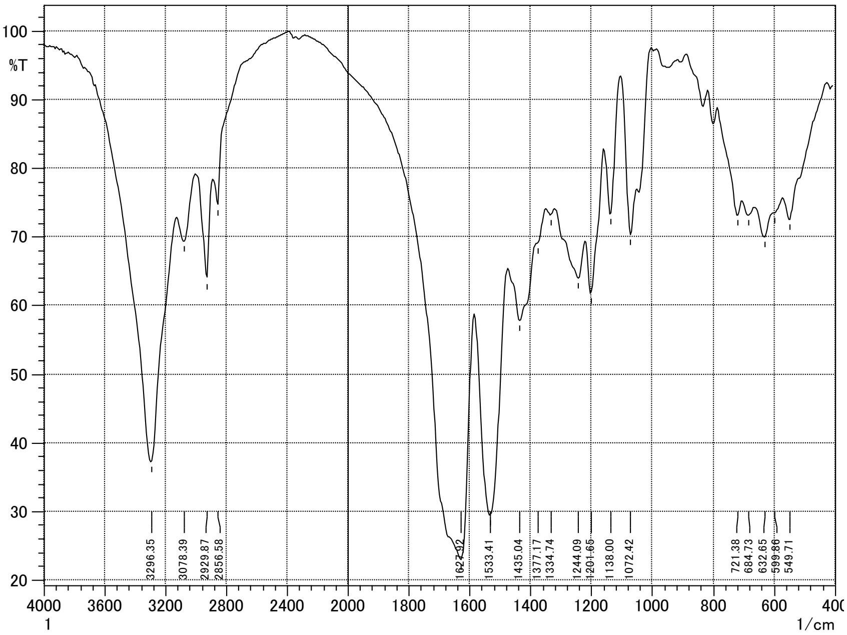


**Figure S12:** FTIR spectrum of $(SG)_5$-KSS-$K(C_{10}H_{21})_2$ **4**.
